# Testicular Polyarteritis Nodosa Mimicking Testicular Neoplasm

**DOI:** 10.1100/tsw.2010.194

**Published:** 2010-10-01

**Authors:** Gokhan Atis, Omer Faruk Memis, Hasan Samet Güngör, Ozgur Arikan, Yesim Saglican, Turhan Caskurlu

**Affiliations:** ^1^ Göztepe Training and Research Hospital, 2nd Urology Clinic, Istanbul, Turkey; ^2^ Nisantasi Pathology Laboratory, Istanbul, Turkey

**Keywords:** polyarteritis nodosa, testis, vasculitis

## Abstract

Polyarteritis nodosa (PAN) presents mostly as a systemic disease characterized by necrotizing vasculitis affecting small- and medium-sized arteries. Rarely, the inflammatory process is isolated and involves a single organ without systemic manifestations. We described the case of a 57-year-old patient with isolated testicular PAN who presented with a testicular mass mimicking a primary testicular tumor.

## INTRODUCTION

Polyarteritis nodosa (PAN) is a systemic necrotizing vasculitis that affects small- and medium-sized arteries. PAN can affect multiple organs, including the skin, kidneys, and gastrointestinal tract. Isolated forms of PAN without evidence of widespread disease are very rare. We describe a patient with isolated PAN of the testis who presented with a testicular mass mimicking a primary testicular tumor. The final diagnosis of isolated testicular vasculitis was established following histological examination of the orchidectomy specimen.

## CASE REPORT

A 57-year-old patient presented with a 10-day history of right testicular pain. On physical examination, the right testis was swollen and a mass lesion was palpated at its upper pole. Scrotal ultrasound examination revealed a hypoechoic, heterogenic, intratesticular mass that was about 3 cm in diameter with no evidence of invasion of the tunica albuginea (Fig. 1). Full blood count, serum electrolytes, liver function tests, tumor markers [AFP= 1.2 IU/L (N: <5 IU/L), β-HCG = 2.5 IU/L (N: <5 mIU/ml), LDH 76 IU/L (N: <243 IU/L)] were all within the normal range. Chest radiography and abdominal computed tomography were normal.

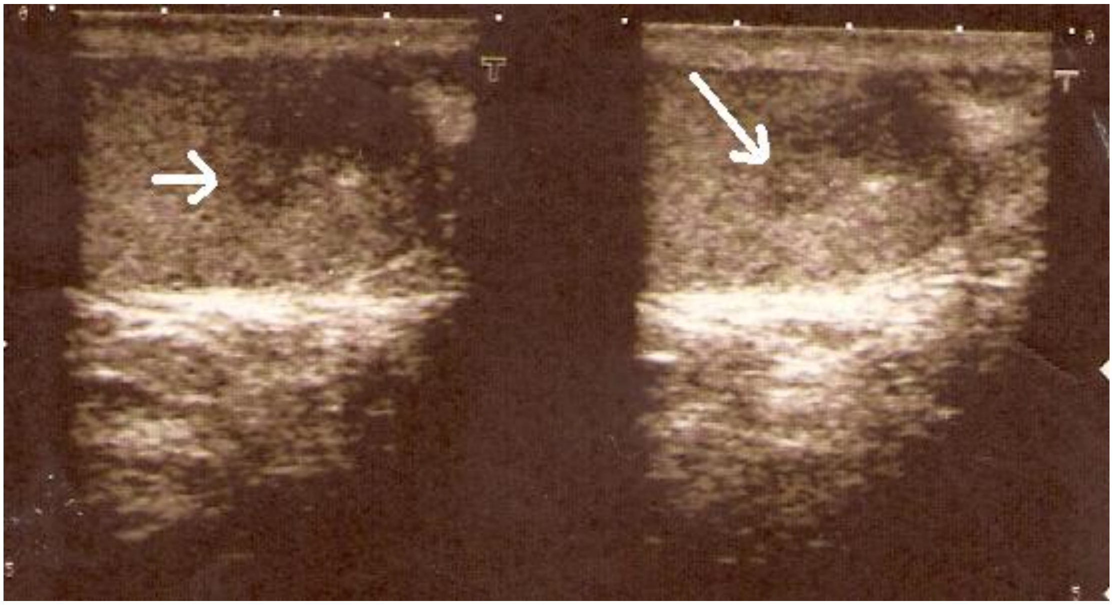

**FIGURE 1.** Doppler ultrasound image of the right testis. A nonvascularized, heterogeneous, hypoechoic, intratesticular mass is visible.

The patient underwent radical orchidectomy, where histopathology revealed multiple hemorrhagic areas occupying most of the testicular volume and necrotizing vasculitis in the middle-sized arteries that suggested PAN. No evidence of malignancy was found.

Postoperative laboratory evaluation, including erythrocyte sedimentation rate, cytoplasmic antineutrophil cytoplasmic antibodies (ANCA), perinuclear ANCA, rheumatoid factor, tests for hepatitis C virus, and hepatitis B surface antigen, were all normal or negative. Despite the absence of systemic manifestations of disease, these results led to a diagnosis of isolated testicular PAN.

## DISCUSSION

PAN is a systemic vasculitis characterized by necrotizing inflammation of medium- and small-sized arteries. PAN was described by Kussmaul and Maier in 1866 and has been known to affect multiple organs, including the skin, kidneys, gastrointestinal tract, and peripheral and central nervous systems, since isolated arteritis was seen in the appendix, which was reported by Plaut in 1932. Isolated disease has been observed also in the gall bladder, uterus, skin, lungs, breast, and kidneys[1,2].

The pathogenesis of PAN is not well understood. Most cases are idiopathic, but may be associated with immune complex deposition. There is an association between classic PAN with hepatitis B virus infection in 7–10% of the cases. With the advent of HBsAg vaccination, the disease is becoming more rare[3,4].

Testicular involvement occurs in up to 18% of patients with systemic PAN. At autopsy, this system is involved in 60–86% of cases[5,6]. Conversely, symptomatic vasculitis confined to the testis without clinical or laboratory evidence of systemic disease is extremely rare. According to our knowledge, only 13 isolated testicular PAN cases have been reported[7].

Definitive diagnosis of isolated PAN requires pathological examination by orchidectomy or biopsy. However, biopsy is contraindicated in suspicous testicular masses. In our case, radical orchidectomy was performed because of the suspicious testicular mass. Pathological examination showed focal testicular infarcts and recent bleeding, consistent with a diagnosis of PAN (Fig. 2). Cross-sections of the spermatic cord indicated necrotizing vasculitis of the spermatic artery and veins (Fig. 3).

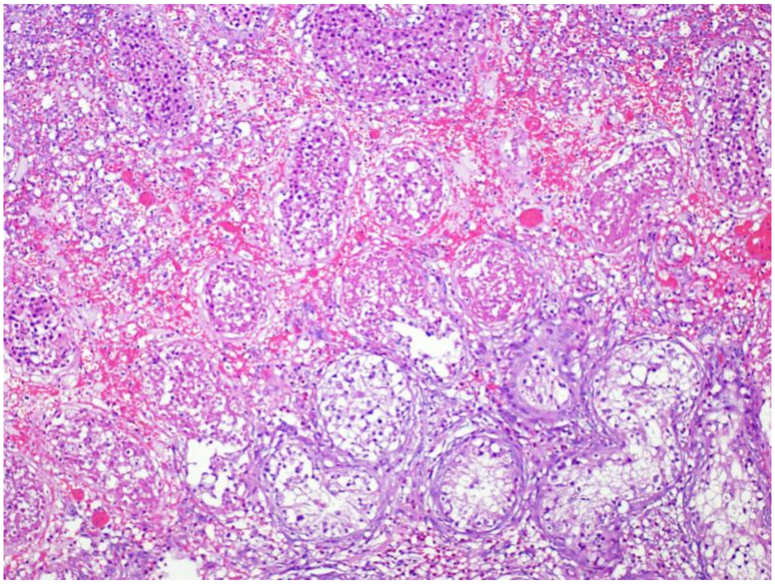

**FIGURE 2.** Histopathological image with hematoxylin and eosin stain of right testis (original magnification ×100). Focal testicular infarcts and recent bleeding are seen.

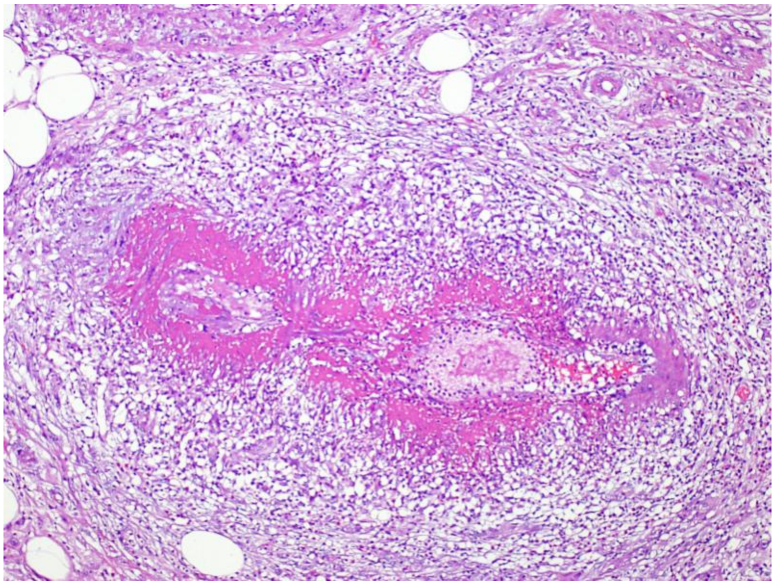

**FIGURE 3.** Histopathological image with hematoxylin and eosin stain of cross-sections of the spermatic cord reveals necrotizing vasculitis of the spermatic artery and veins (original magnification ×200).

There is no consensus regarding the treatment of isolated forms of PAN, although it is postulated that the excision of the affected organ is curative with no need for pharmocologic therapy. Our patient has remained asymptomatic after orchidectomy, with no evidence of systemic vasculitis. The complete absence of systemic symptoms and normal laboratory results suggest no need for further invasive diagnostic procedures, such as renal, skin, or muscle biopsies[2,8].

In conclusion, the differential diagnosis of scrotal painful mass includes torsion of the testis, appendicular torsion, epididymitis, trauma, infectious orchitis, and neoplastic processes[2]. In spite of the fact that management will not change, PAN should be included in the differential diagnosis of a testicular mass.

## References

1. Plaut, A. (1932) Focal arteriolitis. *Am. J. Pathol*. **8,** 620–621.
2. Navarro, H.P., Viana, L.B., Moreno, M.J.D., et al. (2007) Isolated testicular polyarteritis nodosa. *Urology* **70,** 178.e7–e8.
3. Guillevin, L., Lhote, F., Cohen, P., et al. (1995) Polyarteritis nodosa related to hepatitis B virus. A prospective study with long-term observation of 41 patients. *Medicine (Baltimore)* **74,** 238–253.
4. Guillevin, L., Mahr, A., Callard, P., et al. (2005) Hepatitis B virus-associated polyarteritis nodosa: clinical characteristics, outcome, and impact of treatment in 115 patients. *Medicine (Baltimore)* **84,** 313–322.
5. Persellin, S.T. and Menke, D.M. (1992) Isolated polyarteritis nodosa of the male reproductive system. *J. Rheumatol*. **19,** 985–988.
6. Shurbaji, M.S. and Epstein, J.I. (1988) Testicular vasculitis: implications for systemic disease. *Hum. Pathol*. **19,** 186.
7. Tanuma, Y., Oda, T., Yokoo, A., et al. (2003) Recurrent polyarteritis nodosa limited to the testis. *J. Urol*. **170,** 1953.
8. Huisman, T.K., Collins, W.T., and Voulgarakis, J.R. (1990) Polyarteritis nodosa masquerading as a primary testicular neoplasm: a case report and review of the literature. *J. Urol*. **144,** 1236–1238.
